# Pre-slaughter, slaughter and post-slaughter practices of slaughterhouse workers in Southeast, Nigeria: Animal welfare, meat quality, food safety and public health implications

**DOI:** 10.1371/journal.pone.0282418

**Published:** 2023-03-03

**Authors:** Emmanuel O. Njoga, Stanley U. Ilo, Obichukwu C. Nwobi, Onyinye S. Onwumere-Idolor, Festus E. Ajibo, Chinwe E. Okoli, Ishmael F. Jaja, James W. Oguttu

**Affiliations:** 1 Department of Veterinary Public Health and Preventive Medicine, Faculty of Veterinary Medicine, University of Nigeria, Nsukka, Nigeria; 2 Department of Animal Science, Faculty of Agriculture, University of Nigeria, Nsukka, Nigeria; 3 Department of Animal Production, Faculty of Agriculture, Delta State University of Science and Technology, Ozoro, Delta State, Nigeria; 4 Department of Animal Health and Production, Enugu State Polytechnic, Iwollo, Nigeria; 5 Department of Veterinary Public Health and Preventive Medicine, Faculty of Veterinary Medicine, University of Abuja, Abuja, Nigeria; 6 Department of Agriculture and Animal Health, College of Agriculture and Environmental Sciences, University of South Africa, Johannesburg, South Africa; University of Ilorin, NIGERIA

## Abstract

**Background:**

Pre-slaughter stress or the welfare condition of food-producing animals (FPAs) and the slaughter practices of slaughterhouse workers (SHWs) are critically important for the safety and quality of meats processed in slaughterhouses (SHs). Consequently, this study determined the pre-slaughter, slaughter, and post-slaughter (PSP) practices of SHWsin four SHs in Southeast, Nigeria; and discussed the impacts on meat quality and safety.

**Methods:**

The PSP practices were determined by observation method. Additionally, a structured and validated closed-ended questionnaire was used to determine the knowledge of the SHWs on: the effects of poor welfare (preslaughter stress) on the quality and safety of meats produced, carcass/meat processing practices and modes of transmission of meat-borne zoonotic pathogens during carcass/meat processing. Finally, a systematic post-mortem inspection (PMI) was conducted on cattle, pigs and goats slaughtered, and economic losses accruable from condemned carcasses/meats were estimated.

**Results:**

Food-producing animals were transported to the SHs or held in the lairage under inhumane conditions. A pig being conveyed to one of the SHs was seen gasping for air, as it was firmly tied on motorbike at the thoracic and abdominal regions. Fatigued cattle were forcefully dragged on the ground from the lairage to the killing floor. Cattle for slaughter were restrained, held in lateral recumbency and left groaning, due to extreme discomfort, for about one hour before slaughter. Stunning was not performed. Singed pig carcasses were dragged on the ground to the washing point. Although more than 50% of the respondents knew the modes of transmission of meat-borne zoonotic pathogens during meat processing, 71.3% of the SHWs processed carcasses on bare floor, 52.2% used same bowl of water to wash multiple carcasses while 72% did not wear personal protective equipment during meat/carcass processing. Processed meats were transported to meat shops in an unsanitary conditions, using open vans and tricycles. During the PMI, diseased carcasses/meats/organs were detected in 5.7% (83/1452), 2.1% (21/1006) and 0.8% (7/924) of the cattle, pig and goat carcasses inspected, respectively. Gross lesions pathognomonic of bovine tuberculosis, contagious bovine pleuro-pneumonia, fascioliasis and porcine cysticercosis were detected. Consequently, 391,089.2 kg of diseased meat/organs valued at 978 million Naira (235, 030 USD) were condemned. There were significant associations (p < 0.05) between educational level and the use of personal protective equipment (PPE) during slaughterhouse operations and knowledge that FPAs can harbour zoonotic pathogens (p = < 0.001) transmissible during carcass processing. Similarly, significant association was observed between working experience and use of PPE; and between geographical location of the respondents and knowledge that zoonotic pathogens in animals are transmissible during carcass processing or via the food chain.

**Conclusion:**

The findings show that slaughter practices of SHWs have detrimental impacts on the quality and safety of meats processed for human consumption in Southeast, Nigeria. These findings underscore the need to: improve the welfare condition of slaughter-animals, mechanise abattoir operations, train and retrain the SHWs on hygienic carcass/meat processing practices. There is a need to adopt strict enforcement of food safety laws to promote meat quality, food safety and consequently promote the health of the public.

## Introduction

Consumption of animal proteins (APs), particularly meats, is an essential part of the human food culture globally. Meat is important in human diet and nutrition as it contains the nine essential amino acids, particularly tryptophan, threonine and lysine; that are deficient in some plant proteins [1, 2]. To accentuate the importance of APs, the Food and Agricultural Organization of the United Nation (FAO) recommends a minimum per capita daily protein intake of 0.60–0.75 g per kg body weight, of which 60% is expected to be of animal origin [3, 4]. Beef, chicken, pork, mutton and chevon are the most consumed meat types in Nigeria [5, 6]. Of the estimated 1.45 million tons of meats consumed in Nigeria in 2020, 360,000 tons were beef; representing about half of the beef consumed in the entire West African countries [7]. To promote meat hygiene/food safety and protect public health in Nigeria, the recently promulgated Animal Diseases (Control) Act, 2022 seeks to prevent the spread of animal diseases, especially zoonoses. Pursuant to this law, meats from food-producing animals (FPAs) are expected to be processed in a slaughter facility, inspected and certified disease free by Veterinarians before being released for human consumption. Therefore, the slaughter facilities play critical roles in the prevention, detection, control and eradication of zoonotic, infectious and contagious animal diseases.

Slaughter facilities are approved and registered premises for ante-mortem inspection and humane slaughter of FPAs, systematic inspection of meats/carcasses, processing of meat and preservation/storage of meats/meat-products. Depending on the availability of slaughter and meat processing equipment, slaughter facilities can be classified as slaughter slabs (having little or no facilities and usually situated in rural areas), slaughterhouses [SHs] and abattoirs (having modern carcass processing equipment, mechanised and mostly found in urban settings) [8, 9]. As at 2008, there were 1,239 slaughter facilities in Nigeria, which consisted of 30 abattoirs, 132 SHs and 1077 slaughter slabs [8, 10]. However, most of the abattoirs and SHs are now dilapidated and in a state of disrepair [9–13]. The primary role of slaughter facilities is to ensure hygienic processing and release of safe, quality and wholesome meat to the public; through efficient ante-mortem and post-mortem inspections, re-inspection of dressed meats at sales outlets, and condemnation and subsequent destruction of unfit meats/animal products [10, 12, 13].

In order to effectively perform these crucial food safety roles, the pre-slaughter, slaughter and post-slaughter (PSP) practices of the slaughterhouse workers (SHWs), as well as their perceptions towards food safety/meat hygiene and knowledge of modes of transmission of zoonotic meat-borne pathogens at the SHs, are vital. For instance, poor pre-slaughter handling of animals awaiting slaughter or inhumane slaughter practices has tremendous effects on the welfare/physiology of the animals as well as the physiochemical and sensory qualities of the meats [14, 15]. Exhaustion due to stress or inhumane handling of FPAs prior to slaughter can cause depletion of the tissue glycogen and give rise to carcass/meat defects (poor meat quality) [16–18]. Major defects in meat processing industries include Pale, Soft and Exudative (PSE) meat, Dark, Firm and Dry (DFD) meat and poor water holding capacity (WHC) of meat [19, 20]. These defects negatively affect blooming, sensory characteristics (tenderness, juiciness, flavour, taste and colour) and other indices of meat quality; which detrimentally affect profitability in the meat production industry [21]. Economic losses due to meat defects or quality issues, particularly DFD and PSE, in the UK were estimated at 30 million USD annually [22]. Tarrant and co-researchers [23] estimated that DFD meat defect alone costs the UK about nine million Pounds yearly while bruising diminishes profitability in the country’s meat industry by an additional five million pounds annually.

In addition to meat defects, the hygienic status and safety of dressed meats are also paramount to consumers. Poor animal welfare (AW) and stress factors play cardinal roles in meat/food safety. An animal enjoys good welfare if scientifically proven to be free from thirst, hunger and malnutrition, discomfort and pain, injury and disease, fear and distress; and therefore is able to exhibit its natural behaviours unhindered [24–26]. Prolonged poor welfare or pre-slaughter stress in FPAs can lower their immunity and may cause high tissue cortisol level which facilitates anti-inflammatory and immunosuppressive responses by reducing the production and release of pro-inflammatory cytokine; and enhance their vulnerability to infections with microbial pathogens [27–30]. These pathogens or diseases they cause may be zoonotic and, therefore transmissible to humans especially through the food chain [31, 32]. Stress-induced vasoconstriction may lead to poor bleed-out during slaughter and consequent retention of zoonotic haematogenous infections in processed meats [33, 34]. Furthermore, the sanitary condition of the slaughter facility, zoo-sanitary status of FPAs, and the attitude/perceptions of SHWs towards food safety/meat hygiene are essential for production and sale of safe, quality and wholesome meats for human consumption.

In view of the plethora of antithetical and repugnant effects of unhygienic carcass/meat processing practices and poor AW on meat safety/quality, this study assessed the PSP practices of SHWs, conducted post-mortem inspection (PMI) of carcasses/meats and evaluated the sanitary situation of slaughter facilities in Southeast, Nigeria. The study also ascertained the perception of SHWs on hygienic meat processing practices and their knowledge on modes of transmission of zoonotic meat-borne infections during routine slaughterhouse operations. This study has become necessary because published information on the PSP practices in Southeast, Nigeria is few and far between. The findings of the present study, among other things, provide the current status of PSP practices in the study area, highlight the meat quality and food safety implications. Furthermore, findings reported here can guide policy formulation to improve the safety and quality of meats processed for human consumption in Southeast, Nigeria.

## Materials and methods

### The study area

**Fig 1:**
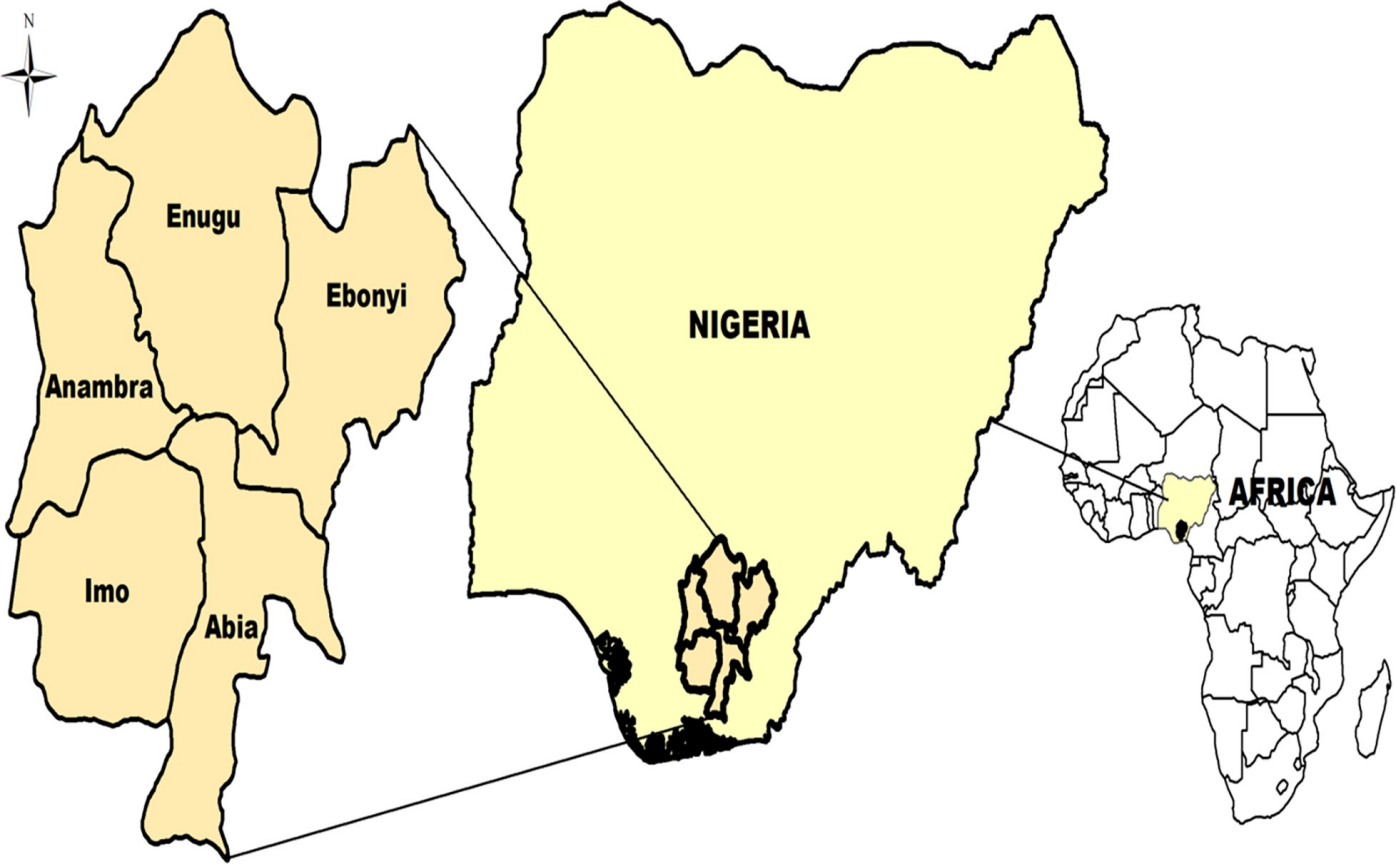
Map of Nigeria highlighting the Southeast geopolitical zone surveyed in this study.

In Nigeria, the Southeast is one of the country’s six geographical and political zones/regions. The region comprises Abia, Anambra, Ebonyi, Enugu and Imo States (Fig 1). Located on latitude 5° 45’ 00" N and longitude 8° 30’ 00"E, the zone has a total land mass of about 40,000 km^2^. The Southeast zone borders the River Niger on the West, the Niger Delta on the South, the North Central zone on the North, and Cross River State on the East. Aba and Enugu are the most populated south-eastern metropolises, being the tenth and fourteenth most populous cities in Nigeria [35]. With an estimated population of about 45 million, the Southeast contributes more than one-fifth of Nigeria’s 216 million people [35]. There are 218 slaughter facilities (majority are slaughter slabs) in the Southeast with an annual combined slaughter capacity of approximately 900,000 animals (cattle, pigs and goats only) [8].

### Study design and sampling procedure

**Fig 2:**
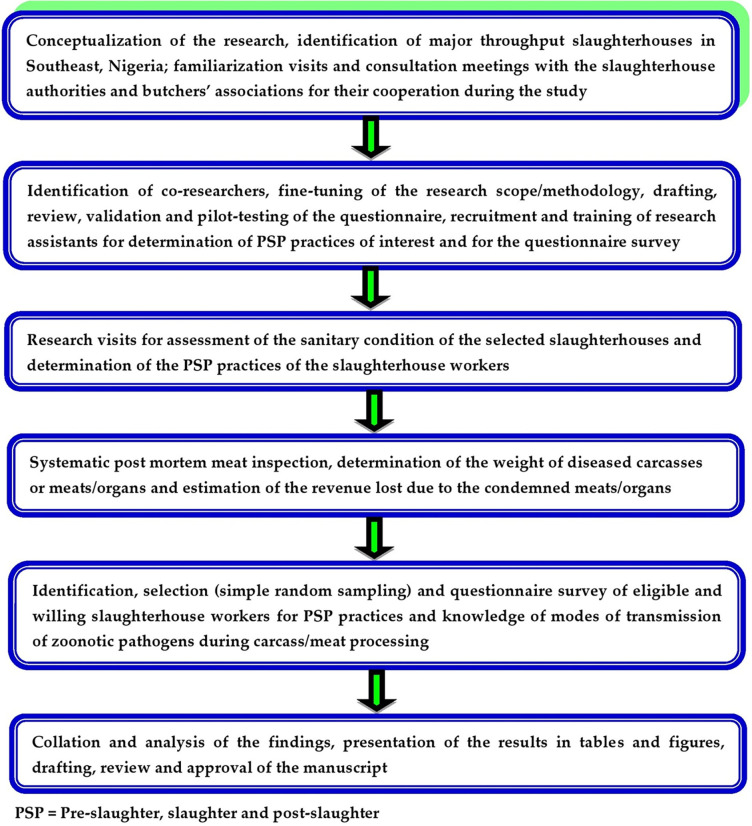
Diagrammatic illustration of the study methodology from conceptualization to manuscript writing.

An overview of the methodology of this study is presented in Fig 2. A cross-sectional study design was adopted. Data were collected by direct observation method, PMI, and a standard questionnaire. Four majorSHs across three of the five States in the study area were purposively selected for the study. The SHs were Akwata (Enugu State), Ikpa (Enugu State), Kwata (Anambra State), and Central meat market (Ebonyi State). These SHs process about 65% of cattle, pigs, and goats slaughtered in all the slaughter facilities in the Southeast [8].

### Determination of the pre-slaughter, slaughter, and post-slaughter practices

Each selected slaughterhouse was visited fortnightly, particularly on Saturdays (when a large number of FPAs are usually slaughtered), for six months. During the visit, the sanitary condition of the SHs and the PSP practices of the SHWs processing cattle, pig and goat carcasses were assessed by the direct observational study technique as described by Holmes [36]. Some of the PSP practices ascertained were transportation condition of FPAs sent for slaughter, welfare status of animal awaiting slaughter, restraint method and the overall handling method during slaughter, carcass/meat processing; and the manner of transporting processed meats to sales outlets. Poor handling and cruel practices were documented.

### Post mortem inspection and economic loss estimation

Thorough PMI of carcasses/meats (head, pluck, organs and muscles) was conducted as described by Biffa et al. [37] and Woldemariyam et al [38] to determine the presence of lesions of common zoonotic or economically important livestock diseases such as bovine tuberculosis, contagious bovine pleuro-pneumonia, porcine cysticercosis, contagious caprine pleuro-pneumonia and fascioliasis. A minimum sample size of 385 for each of the animal/carcass types was computed for the purposes of PMI study, using the Raosoft^®^ sample size calculator freely available online at http://www.raosoft.com/samplesize.htm. The sample size computation assumed 50% prevalence (since there is no report on the prevalence of the lesions in the Southeast Nigeria, to the best of our knowledge), 5% error margin and 95% confidence level. Carcasses inspected were randomly selected. Systematic sampling was used to select one in every five carcass (for each species: cattle, pigs or goats). However, the first carcass for each FPA type to be inspected was selected by simple random sampling (toss of coin, the display of head meant yes–to be sampled). Each selected carcass was then subjected to PMI. The PMI protocol involved visual inspection, palpation and incision of organs/tissues (massetter muscle, tongue, lungs, tracheal, liver, heart, intercostal muscle, spleen and the kidneys). Also, the retropharyngeal, tracheobronchial, mediastinal, mandibular, parotid and prescapular lymph nodes were palpated, longitudinally incised and inspected. Diseased carcasses/organs/meats were weighed and wholly or partially condemned, depending on the severity of the lesion. Whole carcass condemnation involves total condemnation while partial condemnation means passing the carcass as fit for human consumption only after the observed lesions were trimmed-off, or the carcasses were subjected to further treatment. Economic losses associated with the condemned meats were estimated based on the weight of the condemned meant and the prevailing average market prices of beef, pork, and chevon (2500 Naira/ 6 USD) in the study area. The monetary values were calculated in Nigerian Naira and converted to US dollar based on the official exchange rate of 416 Naira per US dollar.

### Data collection

#### Questionnaire survey

A structured, validated and pilot-tested closed-ended questionnaire was used to ascertain the perception of the SHWs about hygienic meat processing practices as well as their knowledge of modes of spread of meat-borne zoonotic pathogens during carcass/meat processing. The questionnaire consisted of 25 questions and included: socioeconomic characteristics of the respondents (eight questions), meat hygiene/food safety practices during carcass/meat processing (10 questions), and knowledge of modes of transmission of zoonotic pathogens during meat processing (7 questions). A copy of the questionnaire is attached as supporting information (S1 Table). The raw data on socio-demographics, perceptions and knowledge of the slaughterhouse workers on food safety practices and modes of transmission of meat-borne zoonotic is also presented in table as supporting file (S2 Table).

The questionnaire was first subjected to face and content validations [39]. Afterwards, a three-man panel of specialists critically reviewed the 25 questions in the questionnaire, and scored each question on a 4-poin scale based on its relevance and clarity, and suggested changes where necessary. The average scores for relevance and clarity were calculated and used to compute the scale-cumulative validity index (s-CVI) and item-cumulative validity index (i-CVI) as described by Zamanzadeh et al. [40]. Questions with s-CVI and i-CVI values ≥ 0.9 were accepted. But questions with s-CVI and i-CVI values < 0.9, were changed based on changes recommended or suggested by the experts/reviewers to enhance their clarity or relevance. Thereafter, the questionnaire was pilot tested on 25 respondents before the actual survey, and inconsistencies were corrected. Next, Cronbach’s Alpha test was performed on IBM^®^ SPSS version 25 (SPSS Inc., Chicago, Illinois, USA). This generated an alpha-value of 0.79 (> the 0.7 benchmarks), which further indicated the validity and reliability of the questionnaire in obtaining the parameters of interest. The reliability of the questionnaire was further confirmed by the test-retest method [41].

A total of 157 respondents from a sampling frame of 392 (determined by the head count of all SHWs who reported to work on the day of the questionnaire survey) were randomly selected and surveyed. The inclusion criteria included: being ≥ 18 years of age, willingness to partake in the survey and working in any of the four selected SHs. After applying the inclusion criteria, 157 respondents were selected from 308 eligible participants by simple random sampling (toss of coin, display of head meant YES—to be interviewed). The respondents were assured of the confidentiality of their responses. Care was taken to ensure that each selected respondent was surveyed just once. The survey adopted an interview schedule format in accordance with the Helsinki Declaration of the World Medical Association in 2013 [42]. The questionnaire content was translated in the native language for the few respondents who had limited proficiency in the English language. Participation in the questionnaire survey was entirely voluntary and at the discretion of the eligible SHWs. Personal identification information of the respondents surveyed was not collected.

### Data analysis and presentation

Information on the PSP practices and PMI were presented in tables and figures. Data from the completed questionnaire were collated and analysed—descriptive and inferential statistics. Fishers’ exact test was used to check for significant (p < 0.05) association between socio-demographics (educational level, working experience, and geographical location) of SHWs and hygienic carcass/meat processing practices and knowledge of modes of transmission of meat-borne zoonotic pathogens during slaughterhouse operations. The statistical significance was set at 5% probability level using IBM^®^ SPSS statistics version 25 (SPSS Inc., Chicago, Illinois, USA).

### Ethical approval

Ethical approval (reference number: VPHPM/UNN/22/201) for the study was granted by the Research Ethics Commettee of the Department of Veterinary Public Health and Preventive Medicine, University of Nigeria, Nsukka. Oral informed consent to pertake in the questionnaire study was sought and obtained from the slaughterhouse workers and SHWs associations in the four slaughterhouses surveyed. First, the leadership of the associations, during the farmilarization meetings with the researchers, verbally approved that any interested member of the associations could partake in the survey; and pledged their unanimous support and cooperation to the research team during the course of the study. Additionally, all the 157 willing respondent surveyed gave oral consent to partake in the study (prior to the survey). All the respondents were aged ≥ 18 years and no minor was involved. Only slaughterhouse workers, who were all adults, were surveyed

## Results

### Pre-slaughter, slaughter and post-slaughter practices of slaughterhouse workers

The lairages in the four SHWs surveyed were in very bad conditions. The lairages had no roofs and hence cattle awaiting slaughter were held under the sun’s scorching heat. The animals were overcrowded due to limited spaces in some SHs. Consequently, lacerations/bruises, probably due to horn/fight injuries, were visible on some of the animals. Trucks that conveyed slaughter-cattle to the SHs were overloaded and the animals were dragged down during off-loading at the lairage. During the rainy/wet season, the lairages were usually flooded and the cattle held in muddy non-concrete floor until the day of slaughter. A pig being conveyed to one of the SHs was seen gasping for air, as it was firmly tied on motorbike at the thoracic and abdominal regions. Immobilized or lame cattle destined for slaughter, probably due to transportation-stress or fatigue, were dragged while on lateral recumbency from the lairage to the slaughterhouse floor.

Pigs restrained and conveyed to the SHs were thrown down from motorbikes. Cattle were restrained for slaughter (firmly tied fore and hind limbs), were left groaning under excruciating pains for about 30 minutes before bleeding. The SHWs immobilised slaughter-cattle violently struggling due to the painful killing by stepping on the ventral mandibular region of the animal to reduce aggression. Others were restrained by strangulation/neck twisting to aid bleeding and decapitation; and held in the strangulated position for a while before the slaughter. Animals were bled or decapitated without stunning.

Flaying, evisceration and deboning of carcasses were done on bare (uncovered) slaughterhouse floor and singed pig carcasses were dragged on the ground to the washing point. The slaughterhouse drainage systems were waterlogged with effluents making it suitable breeding grounds for flies as evidence by presence of maggots. Un-evacuated heaps of animal dung, releasing foul smell, and brooding rodents (rats) were seen at Ikpa slaughter. Pregnant cows were among the animals slaughtered for meat and upon evisceration of their uteri, 23 foetuses at various stages of gestation were removed during the PMI.

Processed carcasses/meats were transported to sales outlets on tricycles and open vans used to convey human passengers and hardware materials. At sales outlets, houseflies were perching on meats openly displayed for sale on slabs as buyers compared the weights of various meat cuts with their bare hands.

### Post mortem carcass inspection and estimation of economic losses due to condemnation of diseased organs

**Fig 3:**
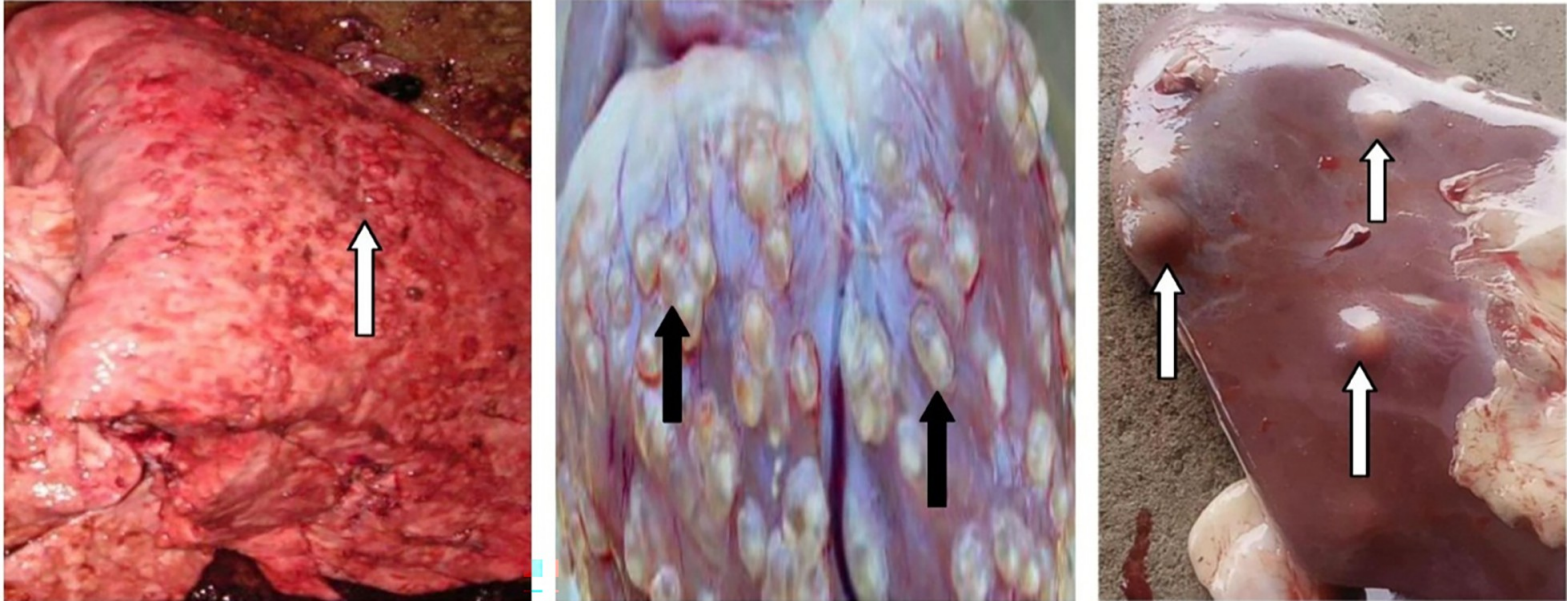
From left to right, bovine lung inundated with nodules suspected to be lesions of tuberculosis, pig heart infested with *Taenia solium* cysticerci and liver showing abscessation, detected during post mortem meat inspection in Southeast, Nigeria.

**Fig 4:**
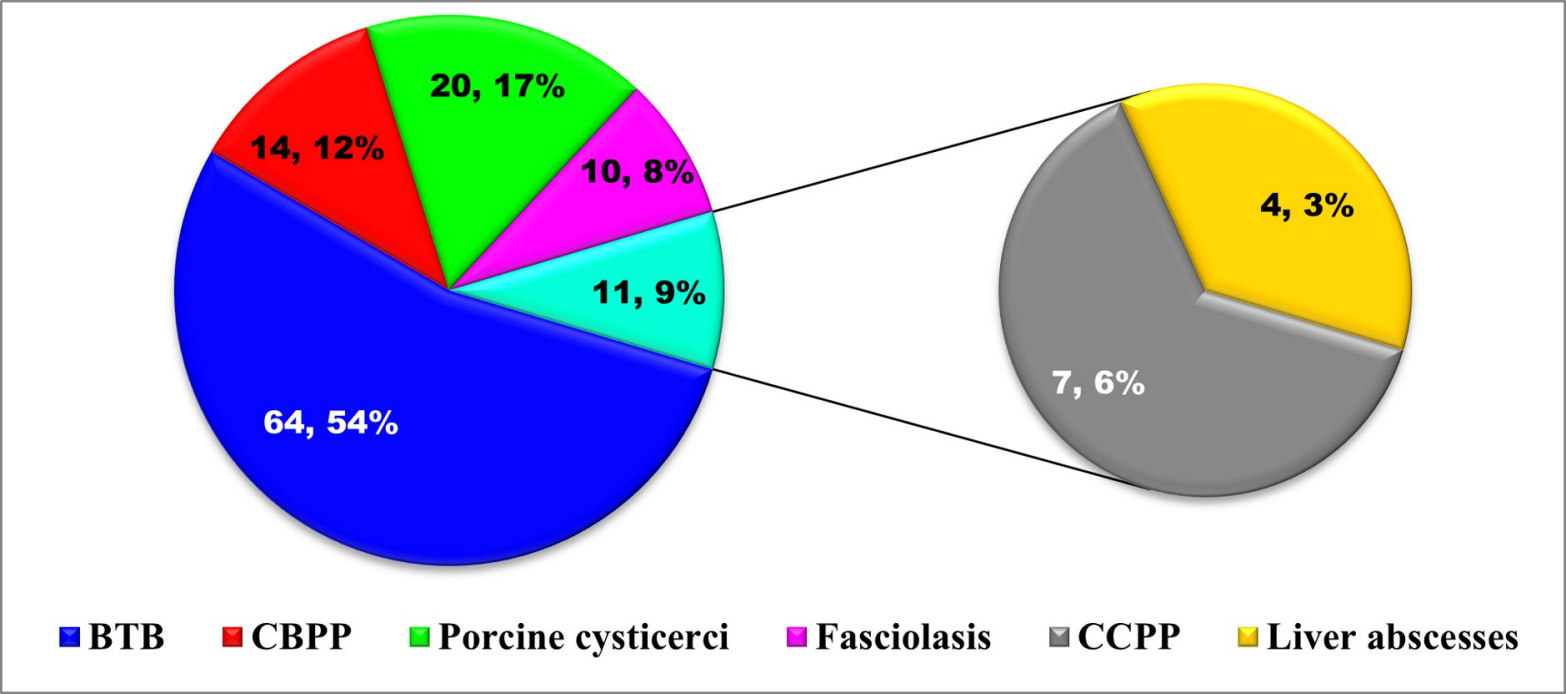
Frequency and proportions of major diseases/lesions (n = 119) found during PMI in four selected slaughterhouses surveyed in Southeast, Nigeria. BTB = bovine tuberculosis, CBPP = contagious bovine pleuro-pneumonia, CCPP = contagious caprine pleuro-pneumonia.

A total of 1452 cattle, 1006 pig and 924 goat carcasses were thoroughly inspected during the six months study. Disease organs were detected in 5.7% (83/1452), 2.1% (21/1006) and 0.8% (7/924) of the inspected cattle, pig and goat carcasses, respectively. Major lesions found were suggestive of bovine tuberculosis, porcine cysticercosis, liver abscesses (Fig 3), contagious bovine pleuro-pneumonia, fasciolasis, and contagious caprine pleuro-pneumonia. The proportions of various diseases/lesions that were found during the PMI in the four selected slaughterhouses surveyed are shown in Fig 4. Eight of the 83 diseased cattle had more than one disease/lesion.

A total of 391, 089.2kg of meats, which consisted of the whole carcass (281,431.7 kg), lungs and trachea (74,127.8 kg), liver (18,689.4 kg), spleen (9,562.7 kg), heart (156.9 kg.) and others (7,120.7 kg) were totally or partly condemned during the six months study. The monetary value of the condemned carcasses/meats was 978 million Naira (USD 235, 030), based on average market price of 2500 Naira (6 USD) per kg of meat in Southeast Nigeria.

### Socio-demographic characteristics

Results on the socioeconomic characteristics of the SHWs surveyed are shown in Table 1. All the workers were males and 62.2% (98/157) were less than 45 years of age. Majority (54.8%, 86/157) were involved in processing cattle carcasses, while 25.5% (40/157) and 19.7% (31/157) were responsible for processing pig and goat carcasses, respectively (Table 1). Majority (47.8%, 75/157) of the SHWs had 10–19 years of working experience. Fourty-five (28.7%, 4/157) of the respondents attained tertiary education, while 11.5% (18/157) had no formal education. Fourty-three(21.7%) of the workers had received formal training on hygienic carcass/meat processing practices. More information on the socio-demographics of the respondents are detailed in Table 1.

**Table 1:** Socioeconomic characteristics of slaughterhouse workers (n = 157) surveyed in Southeast Nigeria.

Variables	Frequency	Proportion (%)
***Gender***		
Male	157	100
Female	0	0
***Age Category***		
< 45 years	98	62.2
≥ 45 years	59	37.6
***Marital status***		
Single	67	42.7
Married	90	57.3
***Occupation***		
Goat carcass processors	31	19.7
Pig carcass processors	40	25.5
Cattle carcass processors	86	54.8
***Slaughterhouse location***		
Anambra State	40	25.5
Enugu State	83	52.9
Ebonyi State	34	21.7
***Working experience in carcass/meat processing***		
< 10 years	46	29.3
10–19 years	75	47.8
≥ 20 years	36	22.9
***Highest educational level attained***		
No formal education	18	11.5
Below tertiary education	94	59.8
Tertiary education	45	28.7
***Had formal training on hygienic/modern carcass/meat processing***		
Yes	34	21.7
No	123	78.3

Table 2 below presents results of the perceptions of SHWs on hygienic meat processing practices and their knowledge on modes of transmission of meat-borne zoonotic pathogens encountered during routine slaughterhouse operations. Only 7% (11/157) of the SHWs practiced stunning prior to bleeding. Pre-slaughter stunning was largely abandoned for various reasons including: religion (56.8%); not knowing that stunning is required prior to bleeding (33.6%) and lack of stunning equipment (9.6%). Majority (93%, 146/157) of the SHWs used water of doubtful microbiological quality for processing carcass/meatand only 22.6% (33/157) of these workers sanitised the water with sanitizer before using it to wash carcasses/meats (Table 2). Majority (71.3%, 112/157) of the SHWs processed/dressed carcass/meat directly on the slaughterhouse floor.; Mean while, 52.2% (82/157) and 28 (44/157) of the respondents used the same water bowl to wash more than one carcass and used PPE during meat processing, respectively (Table 2).

**Table 2:** Perceptions of slaughterhouse workers (n = 157) on hygienic meat processing and their knowledge of modes of transmission of meat-borne zoonotic pathogen during routine slaughterhouse operations in Southeast, Nigeria.

*Variable*	Frequency	Proportion (%)
***Practised stunning before bleeding***		
Yes	11	7
No	146	9
***If you do not stun before bleeding*, *why*?**		
Religious reasons	83	56.8
Lack of stunning equipment	14	9.6
Not aware that stunning is required before bleeding	49	33.6
***Major source/type of water used for carcass/meat processing***		
Well water	40	25.5
Borehole water	85	54.1
Potable water	11	7
Rain water	21	13.4
***If not potable water*, *do you purify the water with water sanitizer before use*?**		
Yes	33	22.6
No	113	77.4
***Do you process carcass or dress meat on bare slaughterhouse floor*?**		
Yes	112	71.3
No	45	28.7
***Do you use same bowl of water or water pool to wash more than one carcass*?**		
Yes	82	52.2
No	75	47.8
***Do you eat or drank while processing carcasses*?**		
Yes	91	58
No	66	42
***If yes*, *do you wash your hands with soap and running water before eating*?**		
Yes	58	63.7
No	33	36.3
***Do you wear personal protective equipment (PPE) while processing carcasses*?**		
Yes	44	28
No	113	72
***Do you eat raw or undercooked meat during carcass processing*?**		
Yes	23	14.6
No	134	85.4
***Can food-producing animals harbour meat-borne zoonotic pathogens*?**		
Yes	81	51.6
No	76	48.4
***Can meat-born zoonotic pathogens in animals spread to humans by handling/processing of infected animals or carcasses or via the food chain*?**		
Yes	111	70.7
No	46	29.3
***Does stress or inhumane handling of animals shortly before slaughter cause poor bleed-out which negatively affects the safety and shelf-life of the processed meats*?**		
Yes	111	70.7
No	463	29.3
***Does stress or inhumane handling of animals awaiting slaughter lower their immunity and increase their susceptibility to meat-borne zoonotic pathogens transmissible during carcass processing or via the food chain*?**		
Yes	67	42.7
No	90	57.3
***Can human transmission of meat-borne zoonotic pathogens result from the use of contaminated water for carcass/meat processing*?**		
Yes	97	61.8
No	60	38.2
***Can non-use of PPE enhance transmission of zoonotic pathogens particularly slaughterhouse workers*?**		
Yes	84	53.5
No	76	46.5
***Does eating/drinking while processing carcass*, *especially with unwashed hands*, *increase your chances of infection with zoonotic pathogens*?**		
Yes	119	75.8
No	38	24.2

Although 51.6% (81/157) of the meat processors knew that FPAs can harbour zoonotic pathogens, only 29.3% (46/157) were aware that these animal pathogens can spread to humans through handling/processing of infected animals/carcasses or via the food chain (Table 2). On the other hand, 57.3% (90/157) did not know that stress or poor welfare conditions, as a result of inhumane handling of animals awaiting slaughter, can lower the animal’s immunity and increase their susceptibility to zoonotic pathogens, which lowers the meat quality (Table 2). However, 53.5% (84/157) of the respondents knew that non-use of PPE can increase the risk of transmission of zoonotic pathogens among occupationally exposed people. A high proportion of SHWs (75.8%, 119/157) were aware that eating/drinking while processing carcass, especially with unwashed hands, may increase their chances of infection with zoonotic pathogens (Table 2).

Educational levels of the respondents was significantly associated with use of PPE (p = 0.022) and awareness that some FPAs can harbour zoonotic pathogens (p = <0.001). However, no association (p = 0.29) was found between educational qualifications and use of same bowl of water/pool of water for washing more than one carcass during meat processing (Table 3). Significant association (p = 0.038) existed between working experience of the SHWs and stunning before slaughter (Table 4). Similarly, significant association was also found between geographical location of the SHWs and knowledge that FPAs can harbour zoonotic infections (p = 0.023), and awareness that these animal infections are transmissible to humans (p = 0.019) (Table 5). Geographical locations of the meat processors had no significant association with stunning (p = 0.957), processing carcasses on the kill floor (p = 0.961), and the use of PPE during meat processing (p = 0.884) (Table 5).

**Table 3:** Association between educational levels of slaughterhouse workers (n = 157) and their hygienic meat processing practices and knowledge on modes of transmission of meat-borne zoonotic pathogen during routine slaughterhouse operations in Southeast, Nigeria.

Variables	Number of YES respondents			P-value
	No formal education (n = 18)	Below tertiary education (n = 94)	Tertiary education (n = 45)	
***Hygienic carcass/meat processing practices***				
Stunned before bleeding	3	4	4	0.141
Dressed carcasses/meats on bare slaughterhouse floor	14	71	27	0.135
Use the same bowl of water or pool of water to wash more than one carcass	10	48	24	0.290
Used of personal protective equipment during abattoir duties	2	23	19	0.022
***Knowledge of modes of transmission of zoonotic meat-borne pathogens during slaughterhouse operations***				
1.	4	38	39	<0.001
2.	10	72	29	0.109
3.	5	70	34	0.001
4.	6	38	23	0.136
5.	8	54	35	0.019
6.	5	45	30	0.028
7.	9	71	39	0.009

1. Know that some food-producing animals can harbour zoonotic meat-borne pathogens

2. Know that stress or inhumane handling of animals shortly before slaughter may cause poor bleed-out, which may enhance retention of meat-borne pathogens negatively affects the shelf-life (keeping quality) of the processed meats

3. Know that some of the zoonotic meat borne pathogens in animals are transmissible to humans by handling/processing infected animals/carcasses or via the food chain

4. Know that stress or inhumane handling of animals awaiting slaughter can lower their immunity and increase their susceptibility to zoonotic meat-borne pathogens transmissible to humans through abattoir operations or via the food chain

5. Know that human infection with zoonotic meat-borne pathogens can result from the use of contaminated water for carcass/meat processing during slaughterhouse operations

6. Know that non-use of personal protective equipment can enhance transmission of zoonotic pathogens among occupationally exposed people, particularly slaughterhouse workers

7. Know that eating/drinking while processing carcass, especially with unwashed hands, may increase your chances of infection with zoonotic pathogens

**Table 4:** Association between working experience of slaughterhouse workers (n = 157) and their hygienic meat processing practices and knowledge on modes of transmission of meat-borne zoonotic pathogen during routine slaughterhouse operations in Southeast Nigeria.

Variable	Number of YES respondents			P-value
	< 10 years (n = 46)	10–19 years (n = 75)	≥ 20 years (n = 36)	
***Hygienic carcass/meat handling practices***				
Stunned before bleeding	3	5	3	0.038
Dressed carcasses/meats on bare slaughterhouse floor	26	59	27	0.028
Use the same bowl of water or pool of water to wash more than one carcass	24	37	21	0.673
Used of personal protective equipment during abattoir duties	8	20	16	0.024*
***Knowledge of modes of transmission of zoonotic meat-borne pathogens during slaughterhouse operations***				
1.	22	41	18	0.748
2.	31	52	28	0.554
3.	25	57	27	0.031
4.	14	29	1	0.154
5.	21	41	28	0.012
6.	18	40	26	0.011
7.	28	61	30	0.019

1. Know that some food-producing animals can harbour zoonotic meat-borne pathogens

2. Know that stress or inhumane handling of animals shortly before slaughter may cause poor bleed-out, which may enhance retention of meat-borne pathogens negatively affects the shelf-life (keeping quality) of the processed meats

3. Know that some of the zoonotic meat borne pathogens in animals are transmissible to humans by handling/processing infected animals/carcasses or via the food chain

4. Know that stress or inhumane handling of animals awaiting slaughter can lower their immunity and increase their susceptibility to zoonotic meat-borne pathogens transmissible to humans through abattoir operations or via the food chain

5. Know that human infection with zoonotic meat-borne pathogens can result from the use of contaminated water for carcass/meat processing during slaughterhouse operations

6. Know that non-use of personal protective equipment can enhance transmission of zoonotic pathogens among occupationally exposed people, particularly slaughterhouse workers

7. Know that eating/drinking while processing carcass, especially with unwashed hands, may increase your chances of infection with zoonotic pathogens

**Table 5:** Association between geographical location of slaughterhouse workers (n = 157) and their hygienic meat processing practices and knowledge on modes of transmission of meat-borne zoonotic pathogen during routine slaughterhouse operations in Southeast, Nigeria.

Variable	Number of YES respondents			P-value
	Anambra (n = 40)	Enugu (n = 83)	Ebonyi (n = 34)	
***Hygienic carcass/meat handling practices***				
Stunned before bleeding	3	6	2	0.957
Dressed carcasses/meat on bare slaughterhouse floor	28	60	24	0.961
Use the same bowl of water or pool of water to wash more than one carcass	25	42	15	0.262
Used of personal protective equipment during abattoir duties	10	24	10	0.884
***Knowledge on modes of transmission of zoonotic meat-borne pathogens during slaughterhouse operations***				
1.	20	50	11	0.023
2.	28	58	25	0.919
3.	23	66	20	0.019
4.	13	34	20	0.067
5.	21	49	27	0.045
6.	20	48	16	0.499
7.	25	65	29	0.055

1. Know that some food-producing animals can harbour zoonotic meat-borne pathogens

2. Know that stress or inhumane handling of animals shortly before slaughter ***may*** cause poor bleed-out, which may enhance retention of meat-borne pathogens negatively affects the shelf-life (keeping quality) of the processed meats

3. Know that some of the zoonotic meat borne pathogens in animals are transmissible to humans by handling/processing infected animals/carcasses or via the food chain

4. Know that stress or inhumane handling of animals awaiting slaughter can lower their immunity and increase their susceptibility to zoonotic meat-borne pathogens transmissible to humans through abattoir operations or via the food chain

5. Know that human infection with zoonotic meat-borne pathogens can result from the use of contaminated water for carcass/meat processing during slaughterhouse operations

6. Know that non-use of personal protective equipment can enhance transmission of zoonotic pathogens among occupationally exposed people, particularly slaughterhouse workers

7. Know that eating/drinking while processing carcass, especially with unwashed hands, may increase your chances of infection with zoonotic pathogens

## Discussion

As observed in this study, the poor welfare conditions FPAs were subjected to during pre-slaughter and slaughter processes at the SHs have deliterious effects on the quality and safety of meats produced, as well as on public health. In the meat processing industries, the problem of poor AW has gone far beyond ethical issues as it affects human health and also the economics and profitability of the entire meat processing value chain [43–49]. Although the pathophysiological mechanism responsible for the development of meat defects and meat deterioration due to poor pre-slaughter AW is complex, it bothers essentially on depletion of tissue glycogen due to exhaustion. Prolonged starvation, dragging, strangulation, excessive beating and other forms of pre-slaughter stress as found in this study enhance fatigue and facilitate depletion of tissue glycogen by enzymatic conversion of glycogen to glucose through the process of glycogenolysis [50]. The presence of sufficient tissue glycogen deposit in FPAs during slaughter is necessary for enzymatic conversion of glycogen to lactic acid, needed to achieve optimal post mortem meat pH values ranging between 5.5 and 5.7 [51–53]. Stressed, fatigued or exhausted animals due to poor welfare conditions will have little or no glycogen reserve for production of lactic acid needed to maintain the meat at the optimum pH value. High post mortem pH values (alkalinity) cannot sufficiently inhibit the activities of spoilage bacteria in the meat [54]. Consequently, this may hasten the meat’s spoilage, deterioration or putrefaction, especially in tropical settings where ambient temperature is usually high and ideal for proliferation of microbial pathogens [55–58]. Therefore, humane handling of FPAs and good AW are essential for optimal post mortem meat pH required to prolong the meat’s shelf life by limiting the proliferation of spoilage bacteria.

Reduced lactic acid production due to poor AW, occasioned by muscle tissue glycogen depletion, also affects the meats’ water holding capacity (WHC) [59]. The WHC refers to the ability of the meat to retain its own moisture upon the application of external force, pressure, or during cooking (heating) [51, 60]. The WHC, is a major determinant of meat quality because drip losses greatly affects some physiochemical and sensory qualities of the meats, including the tenderness, juiciness, blooming (colour) and flavour; and may also lead to formation of meat defects (DFD and PSE) [15, 60–62]. The physiochemical and sensory alterations may lead to down-grading and devaluation of the meats during meat inspection, poor eye appeal at sales outlets and consequently economic losses.

Furthermore, dragging immobilized cattle from the lairage to the slaughter floor increases the levels of creatine phosphokinase (CPK) and aspartate aminotransferase (AST) in meats [63]. These enzyme markers are indicative of muscle damage and low meat quality, due to their adverse effects on meat blooming (colour/appearance), taste, flavour, and other eating qualities [14, 63, 64]. Abnormal changes in the AMP-activated protein kinase-mediated energy metabolism, crosstalk along calcium signalling pathways in the muscle tissues, formation of reactive oxygen species, myofibril protein modifications and cathepsin proteolytic systems are the key biochemical early post mortem changes responsible for poor meat quality and defects [50, 65–67]. These defects may give rise to consumer dissatisfaction and therefore financial losses to the meat producers and the meat industry in general. It was estimated that meat defects (DFD, PSE and bruising) and other wastages in the meat value chain were responsible for loss or wastage of a significant amount of the 330 million tonnes of meat produced globally every year [68–70].

Apart from the problems of poor meat quality and defects, the poor pre-slaughter welfare conditions FPAs are subjected to that were observed in this study, such as transportation stress and holding slaughter-animals in dilapidated lairage under harsh climatic conditions, can lower the immunity of the animals and enhance their susceptibility to zoonotic and economically important livestock diseases. Chronic stress in animals such as poor housing (lairage) condition, starvation/malnutrition and perpetual fear/anxiety due to sights of cruelty and painful killing can inhibit the activities of lymphoid tissues resulting in low immune-cells counts (leucocytopenia), particularly the T-lymphocytes [71–76]. Both physicochemical and psychological types of stress in FPAs may lead to persistent high levels of serum cortisol and corticosteroid, causing increased vulnerability to infectious diseases [77–80]. Exposure of FPAs to stress may also impair the anti-inflammatory functions of the immune system and inhibit the cross-talk of immune cells and signalling networks; resulting in increased susceptibility to infections and diseases; which may be zoonotic or contagious to other animals [81–83]. This can pose significant risk to public health, through consumption of undercooked meats contaminated with commonly endemic meat-borne pathogens such as *Escherichia coli*, *Campylobacter* species and *Staphylococcus* species [84].

The risk of human infection with zoonotic pathogens from FPAs in the study area is of immense public health concern considering that during PMI, gross lesions that are pathognomonic of bovine tuberculosis and porcine cysticercosis were observed in dressed meats. Humans, especially occupationally exposed individuals, are susceptible (via inhalation and ingestion of undercooked infected meat or unpasteurised milk) to both *Mycobacterium bovis* and *Mycobacterium tuberculosis;* the principal aetiological agents of bovine and human tuberculosis, respectively [85–89]. Similarly, humans are also susceptible to cysticerci infection. Humans are the definitive host of *Taenia solium*, and become infected (acquire taeniasis) by ingestion of undercooked pork containing viable cysticerci; while pigs, the intermediate host of *Taenia solium*, acquire the infection (cysticercosis) by consuming viable *T*. *solium* eggs voided in human faeces [90–92]. Occasionally, humans may become the intermediate host of *Taenia solium* by accidental ingestion of the viable egg or gravid proglottid. This may give rise to human cysticercosis and possibly neurocysticercosis, characterized by seizures, epilepsy and other nervous signs [92–95]. Human cysticercosis is the responsible for 30% to 70% of epilepsy cases globally and approximately 80% of the world’s 50 million people suffering from epilepsy live in developing countries [95].

Detection of lesions of zoonotic diseases in processed meats is an indication of poor or lack of PMI procedures in the study area. Carcasses and processed meats should be systematically inspected at the slaughterhouse level and then re-inspected at sales/retail outlets for public health and food safety reasons. However, in most parts of Nigeria, carcass/meat inspection ends at the abattoir level as post-abattoir handling of processed carcasses/meats are largely ignored; giving rise to possible meat adulteration [8, 13]. Lack of qualified man-power may be responsible for poor PMI and the subsequent passing of diseased meats/organs. The 4^th^ schedule of the Nigerian 1999 constitution subsection 1(e) vested the power to oversee the activities of slaughter facilities with Local Government Areas (LGAs). However, some of these LGAs have contracted out the oversight function to individuals and contractors; who have reduced PMI to mere collection of revenues per animal slaughtered. Veterinarians who are well trained on meat inspection and animal disease diagnosis are hardly employed by the individuals/contractors. This could be attributed to the desire to cut cost and maximize economic gains at the detriment of human health; and the ability to detect diseased meats/organs released for human consumption.

The poor hygienic meat processing practices and knowledge of modes of transmission of zoonotic pathogens during carcass processing observed among the SHWs surveyed raises doubts on the safety of meats produced; and further aggravates the possibilities of inter-and-intra species transmission of diseases in the study area. Dressing carcasses/meats directly on slaughterhouse floors, use of water of doubtable microbial quality for meat processing and non-use of PPE during slaughterhouse operations pose great public risks to meat consumers in the study area. However, these findings are not entirely surprising considering that most of the SHWs were not formally trained in carcass/meat processing. Additionally, the hygiene level of the slaughterhouse environment was suboptimal; with dilapidated facilities and crude meat processing methods that could not support production of safe, sound and wholesome meats.

Perhaps, the panacea to these problems includes strict enforcement of meat/food safety or animal disease control laws to improve AW and public health, training and retraining of SHWs on hygienic meat processing practices, provision of basic slaughterhouse amenities, and mechanization of the slaughter facilities. Although Nigeria does not have a stand-alone legislation on AW, the country has enacted some Acts/Code/Laws promoting the welfare of animals and has also amended some obsolete legislation guiding responsible use and ownership of animals. The enactment of the Nigerian meat edict of 1988, the development of the Nigerian Animal Welfare Strategy Framework in 2016 and the recent promulgation of the Animal Disease (control) Act of 2022 are some of the legislative frameworks to improve AW and food safety/public health. These laws proscribe all forms of cruelty, deliberate infliction of pain or suffering to animals; through negligence or failure to act by the animal owners/custodians. The Animal Disease (Control) Act of 2022 provides for the prevention, control, and eradication of zoonotic, infectious, and contagious animal disease to relieve animal suffering and improve public health. However, some acts/Codes/Laws are either not strictly implemented or are completely ignored. This probably explains why AW issues are at the lowest ebbs in Nigeria, especially at SHs surveyed, where the welfare of animals awaiting slaughter are relegated to the background with impunity.

The poor sanitary state of SHs surveyed and the poor hygienic meat processing practices found in this study are in tandem with the findings of other researchers from different parts of Nigeria. The latter observed that carcasses were processed on bare ground, meats were washed with water from the gutters and effluents channelled to nearby natural water bodies, which may be sources of drinking water for the communities around the abattoirs [11, 72, 96, 97]. Outside Nigeria, poor sanitary conditions of SHs and condemnation of meats due to various disease conditions have be reported in South Africa [98, 99], Ethiopia [100], Iran [101], Tanzania [102], Namibia [103] and Kenya [104].

The authors are of the view that operationalizing good AW and slaughter hygienic standards in the SHs surveyed is likely to face difficulties because Nigeria’s meat production and processing sectors are largely controlled by people who deploy obsolete and crude methods of livestock production and meat processing. Consequently, humane slaughter practices and standard hygienic meat processing practices are very rare. These problems are excercebated by the non-availability of mechanized slaughter equipment and certain religious beliefs or personal ideologies that are inimical to AW and food safety. For instance, pre-slaughter stunning have been proven to enhance humane slaughter by greatly reducing pain perception during the slaughter process; but ‘halal’ slaughter, a religious slaughter method widely practiced in the Nigeria, appears to have not fully embraced stunning despite the established AW and food safety benefits [105, 106]. Many researchers have alluded to these facts and reported that reduction of stress/anxiety and pre-slaughter stunning enhanced bleed-out, reduced blood retention in the trachea as well as blood splash in the lungs [50, 107, 108]. The enhanced bleed-out may help to flush out haematogenous infections (which may be zoonotic) and facilitate the prolongation of the shelf life the meat, by reducing the contamination and growth of putrefactive bacteria in the meat. Additionally, improved bleed-out during slaughter reduces tissue cortisol and residual haemoglobin levels in the meat [106, 109, 110]. High tissue cortisol and haemoglobin levels facilitate lipid oxidation and catalyse the activities of autolysis enzymes responsible for onset and progression of PSE meat, particularly in pork [111–114].

Furthermore, the large quantity of condemned meats (391,089.2kg and the estimated economic losses (978 million Naira or 235, 030 USD) is of public health concern. This is particularly appreciated if one considers that protein malnutrition still subsists in some parts of Nigeria, especially among children in rural settings [115, 116]. Therefore such losses are unacceptable and need to be prevented. Moreover, major diseases believed to be responsible for the condemnation of meat, and the consequent humongous financial losses are preventable or can be minimised. Of concern also are the slaughter of pregnant cows and the consequent wastage of bovine foetuses which could have been raised to address the scarcity of AP in the country. If unchecked, these excessive wastages and wanton losses of animal resources may result in an acute shortage and diminution of meat in Nigeria, and concequently worsen the already precarious situation around AP supply in the country [26, 117].

## Conclusion

The pre-slaughter, slaughter and post-slaughter practices of SHWs in Southeast, Nigeria are suboptimal. Similarly, the knowledge of the modes of transmission of meat-borne zoonotic pathogens during routine carcass/meat processing among SHWs is inadequate. Condernmination of diseased carcasses/meats/organs during PMI caused enourmous economic losses during the six months study. Major lesions that lead to the meat condernmination were suggestive of bovine tuberculosis, contagious bovine pleuro-pneumonia, fascioliasis, porcine cysticercosis, contagious caprine pleuro-pneumonia and liver abscesses. The findings of this study raise serious concern on the quality and safety of meats processed for human consumption in the study area and warrant urgent actions to improve animal welfare, meat quality/safety and curtail livestock-associated economic losses in Southeast, Nigeria.

## Recommendations

To remedy the poor PSP practices, at least on the interim, there is need for strict implementation and enforcement of the available laws in Nigeria aimed at protecting animal welfare, food safety and public health; particularly the Meat Edict of 1988 and the recently promulgated Animal Disease (Control) Act of 2022. Those transporting FPAs to slaughter facilities under inhumane conditions could be reprimanded and fined to deter others from doing so. Provision of basic amenities, particularly potable water, in slaughter facilities surveyed and improvement of the sanitary condition of meat processing environment via prompt waste collection, treatment and safe disposal is worthwhile. Training and retraining of SHWs on hygienic meat processing practices and knowledge of modes of spread of the meat-borne zoonotic pathogen is imperative. There is also a need for the employment of Veterinarians, who are professionally trained and certified for meat inspection, to oversee the activities of the SHs. This has potentials to enhance food safety and public health. Finally, upgrading or mechanizing the SHs visited, provision of modern carcass/meat processing equipment and development of internationally acceptable standard operational procedure (SOP) to guide meat processing in slaughterhouses nationwide cannot be over emphasised.

## Supporting information

S1 Table A copy of the questionnaire used in this study. (DOC)

S2 Table Socio-demographics, perceptions and knowledge of the slaughterhouse workers on food safety practices and modes of transmission of meat-borne zoonotic pathogens during carcass processing. (DOC)
