# Supplementary material for: Pre-slaughter, slaughter and post-slaughter practices of slaughterhouse workers in Southeast, Nigeria: Animal welfare, meat quality, food safety and public health implications
Source: PLoS One. 2023 Mar 3;18(3):e0282418. doi: 10.1371/journal.pone.0282418 (PMC9983863; doi:10.1371/journal.pone.0282418)
Supplement: S1 Table — (DOC) [file pone.0282418.s001.doc]

**UNIVERSITY OF NIGERIA, NSUKKA**

**Department of Veterinary Public Health and Preventive Medicine**

Perceptions of slaughterhouse workers on hygienic meat processing and their knowledge on modes of transmission of meat-borne zoonotic pathogen during routine slaughterhouse operations in Southeast, Nigeria

***Please provide your responses by ticking the most appropriate*** option in the response column

| S/n | Questions/information required | Response |
| --- | --- | --- |
| 1. | Gender |  |
|  | Male |  |
|  | Female |  |
| 2. | Age Category |  |
|  | <45 years |  |
|  | ≥ 45 years |  |
| 3. | Marital status |  |
|  | Single |  |
|  | Married |  |
| 4. | Occupation |  |
|  | Goat carcass processors |  |
|  | Pig carcass processor |  |
|  | Cattle carcass processor |  |
| 5. | Slaughterhouse location |  |
|  | Anambra State |  |
|  | Enugu State |  |
|  | Ebonyi State |  |
| 6. | Working experience in carcass/meat processing |  |
|  | < 10 years |  |
|  | 10-19 years |  |
|  | ≥ 20 years |  |
| 7. | Highest educational level attained |  |
|  | No formal education |  |
|  | Bellow tertiary education |  |
|  | Tertiary education |  |
| 8. | Have had formal training on hygienic/modern carcass/meat processing |  |
|  | Yes |  |
|  | No |  |
| 9. | Do you stun slaughter-animals prior to bleeding? |  |
|  | Yes |  |
|  | No |  |
| 10. | If no, why don’t you stun before bleeding? |  |
|  | Religious reasons |  |
|  | Lack of stunning equipment |  |
|  | Not aware that stunning is require before bleeding |  |
| 11. | Major source of water used for carcass/meat processing | |
|  | Well water |  |
|  | Borehole water |  |
|  | Potable water |  |
|  | Rain water |  |
| 12. | If not potable water, do you purify the water with water sanitizer or any other purification method before using the water for carcass processing? |  |
|  | Yes |  |
|  | No |  |
| 13. | Do you process carcass /dress meat on bare slaughterhouse floor? | |
|  | Yes |  |
|  | No |  |
| 14. | Do you use same bowl of water or water pool to wash more than one carcass? | |
|  | Yes |  |
|  | No |  |
| 15. | Do you eat or drink while processing carcasses? |  |
|  | Yes |  |
|  | No |  |
| 16. | If yes, do you wash your hands with soap and running water before eating? |  |
|  | Yes |  |
|  | No |  |
| 17. | Do you use personal protective equipment (PPE) while processing carcasses? |  |
|  | Yes |  |
|  | No |  |
| 18. | Do you consume raw or undercooked meat during carcass processing? |  |
|  | Yes |  |
|  | No |  |
| 19. | Do you know that some food-producing animals can harbour zoonotic pathogens? | |
|  | Yes |  |
|  | No |  |
| 20. | Do you know that stress or inhumane handling of animals shortly before slaughter may cause poor bleed-out which negatively affects the shelf-life of the processed meats? |  |
|  | Yes |  |
|  | No |  |
| 21. | Do you know that some of the zoonotic pathogens in animals are transmissible to humans by handling/processing of infected animals/carcasses or via the food chain? | |
|  | Yes |  |
|  | No |  |
| 22. | Do you know that stress or inhumane handling of animals awaiting slaughter can lower their immunity and increase their susceptibility to zoonotic pathogens transmissible to humans through abattoir operations or via the food chain? | |
|  | Yes |  |
|  | No |  |
| 23. | Do you know that the human infection with zoonotic pathogens can result from the use of contaminated water for carcass/meat processing during slaughterhouse operations? | |
|  | Yes |  |
|  | No |  |
| 24. | Do you know that non-use of PPE can enhance transmission of zoonotic pathogens among occupationally exposed people, particularly slaughterhouse workers? | |
|  | Yes |  |
|  | No |  |
| 25. | Do you know that eating/drinking while processing carcass, especially with unwashed hands, may increase your chances of infection with zoonotic pathogens? | |
|  | Yes |  |
|  | No |  |
