# Supplementary material for: Pre-slaughter, slaughter and post-slaughter practices of slaughterhouse workers in Southeast, Nigeria: Animal welfare, meat quality, food safety and public health implications
Source: PLoS One. 2023 Mar 3;18(3):e0282418. doi: 10.1371/journal.pone.0282418 (PMC9983863; doi:10.1371/journal.pone.0282418)
Supplement: S2 Table — (DOC) [file pone.0282418.s002.doc]

**S2 Table.** Socio-demographics, perceptions and knowledge of the slaughterhouse workers (*n* = 157) on food safety practices and modes of transmission of meat-borne zoonotic pathogens during carcass processing.

| Variables | Frequency | Proportion (%) | |
| --- | --- | --- | --- |
| ***Gender*** |  |  | |
| Male | 157 | 100 | |
| Female | 0 | 0 | |
| ***Age Category*** |  |  | |
| < 45 years | 98 | 62.2 | |
| ≥ 45 years | 59 | 37.6 | |
| ***Marital status*** |  |  | |
| Single | 67 | 42.7 | |
| Married | 90 | 57.3 | |
| ***Occupation*** |  |  | |
| Goat carcass processors | 31 | 19.7 | |
| Pig carcass processors | 40 | 25.5 | |
| Cattle carcass processors | 86 | 54.8 | |
| ***Slaughterhouse location*** |  |  | |
| Anambra State | 40 | 25.5 | |
| Enugu State | 83 | 52.9 | |
| Ebonyi State | 34 | 21.7 | |
| ***Working experience in carcass/meat processing*** |  |  | |
| < 10 years | 46 | 29.3 | |
| 10-19 years | 75 | 47.8 | |
| ≥ 20 years | 36 | 22.9 | |
| ***Highest educational level attained*** |  |  | |
| No formal education | 18 | 11.5 | |
| Bellow tertiary education | 94 | 59.8 | |
| Tertiary education | 45 | 28.7 | |
| ***Had formal training on hygienic/modern carcass/meat processing*** | |  | |
| Yes | 34 | 21.7 | |
| No | 123 | 78.3 | |
| Variable | Frequency | Proportion (%) | |
| Practised stunning before bleeding |  |  | |
| Yes | 11 | 7 | |
| No | 146 | 9 | |
| If you do not stun before bleeding, why? |  |  | |
| Religious reasons | 83 | 56.8 | |
| Lack of stunning equipment | 14 | 9.6 | |
| Not aware that stunning is required before bleeding | 49 | 33.6 | |
| ***Major source/type of water used for carcass/meat processing*** | |  |  |
| Well water | 40 | 25.5 |  |
| Borehole water | 85 | 54.1 |  |
| Potable water | 11 | 7 |  |
| Rain water | 21 | 13.4 |  |
| ***If not potable water, do you purify the water with water sanitizer before use?*** |  |  |  |
| Yes | 33 | 22.6 |  |
| No | 113 | 77.4 |  |
| ***Do you process carcass or dress meat on bare slaughterhouse floor?*** | |  |  |
| Yes | 112 | 71.3 |  |
| No | 45 | 28.7 |  |
| ***Do you use same bowl of water or water pool to wash more than one carcass?*** | |  |  |
| Yes | 82 | 52.2 |  |
| No | 75 | 47.8 |  |
| ***Do you eat or drank while processing carcasses?*** |  |  |  |
| Yes | 91 | 58 |  |
| No | 66 | 42 |  |
| ***If yes, do you wash your hands with soap and running water before eating?*** |  |  |  |
| Yes | 58 | 63.7 |  |
| No | 33 | 36.3 |  |
| ***Do you wear personal protective equipment (PPE) while processing carcasses?*** |  |  |  |
| Yes | 44 | 28 |  |
| No | 113 | 72 |  |
| ***Do you eat raw or undercooked meat during carcass processing?*** |  |  |  |
| Yes | 23 | 14.6 |  |
| No | 134 | 85.4 |  |
| ***Can food-producing animals harbour meat-borne zoonotic pathogens?*** | |  |  |
| Yes | 81 | 51.6 |  |
| No | 76 | 48.4 |  |
| ***Can meat-born zoonotic pathogens in animals spread to humans by handling/processing of infected animals or***  ***carcasses or via the food chain?*** | |  |  |
| Yes | 111 | 70.7 |  |
| No | 46 | 29.3 |  |
| ***Does stress or inhumane handling of animals shortly before slaughter cause poor bleed-out which***  ***negatively affects the safety and shelf-life of the processed meats?*** | |  |  |
| Yes | 111 | 70.7 |  |
| No | 463 | 29.3 |  |
| ***Does stress or inhumane handling of animals awaiting slaughter lower their immunity and increase their susceptibility to meat-borne zoonotic pathogens transmissible during carcass processing or via the food chain?*** | |  |  |
| Yes | 67 | 42.7 |  |
| No | 90 | 57.3 |  |
| ***Can human transmission of meat-borne zoonotic pathogens result from the use of contaminated***  ***water for carcass/meat processing?*** | |  |  |
| Yes | 97 | 61.8 |  |
| No | 60 | 38.2 |  |
| ***Can non-use of PPE enhance transmission of zoonotic pathogens particularly slaughterhouse workers?*** | |  |  |
| Yes | 84 | 53.5 |  |
| No | 76 | 46.5 |  |
| ***Does eating/drinking while processing carcass, especially with unwashed hands, increase your chances of infection with zoonotic pathogens?*** | |  |  |
| Yes | 119 | 75.8 |  |
| No | 38 | 24.2 |  |
